# Monthly Summary

**Published:** 1872-06

**Authors:** 


### MONTHLY SUMMARY.

*Transformation of Medulla into Bone.*—M. Demarquay lately presented to the *Academie de Medicine* an interesting example of ossification of the medulla of the left humerus, resulting from a gun-shot fracture received nearly a year before—namely, on the second of January, 1871. At the time of admission of the patient, the case was considered favorable for conservation of the limb, but under treatment union did not take place, and in the month of June following not only had numerous abscesses formed upon the limb, but it had considerably increased in size along its entire extent, at the same time that numerous fistulous openings, leading through a thick layer of new osseous tissue, rendered easy the detection of necrosed bone in its interior. The arm being disarticulated and the humerus sawn along its length, it was found that the layer of new bone by which it was surrounded presented numerous spaces communicating with the dead bone, within which comprised the entire thickness of the humerous, considerably diminished in volume, while the extremity and circumferance of the medulla had undergone osseous transformation, the central portion being destoyed. The circumstance was interesting as showing that ossification of the medulla may take place, a fact which has from the most ancient time been successively accepted and denied by physiologists, but lately proved by Ollier, although only in a pathological condition. On comparing the ossification of the medulla with bony formations furnished by the periosteum, we observe the small formative power of the former as compared with the latter when separated from mortified bone; the periosteal bone has a considerable volume, while the ossified medulla is thin and fragile. M. Demarquay observed that in the specimen before him the transformation was in no way mere calcification of the medulla, but veritable ossification taking place in the medullary membrane as described by Bichat; and if the present facts have not demonstrated the existence of that membrane they have at least shown that the elements of the circumferance of the medulla are alone capable of transformation under the influence of long-continued irritation.—*The Doctor.*

*Increase of Heart Disease.*—An evil recognized is sometimes half cured; and the intellectual classes, looking at figures such as those Dr. Quain has displayed in his interesting Lumleian Lectures, at the College of Physicians, on "Diseases of the Walls of the Heart," may well consider the propriety of attending to the hygiene of their lives, as well as of their houses; and remember that, to enjoy and benefit by even pure air, soil and water, they must avoid disabling heart and brain by the incessant labors which too often make useful lives joyless, and embitter the harvesting of the crop which has been too diligently sown. These warning figures tell that, during the last twenty years, the total of deaths of males at all ages from heart disese, has increased in number from 5,746 in 1851, to 12,428 in 1870. The percentage of deaths from heart disease for 1,000 of population living was 755 between the years 1851 and 1856; it has risen to 1,085 from 1866 to 1870. This increase, it must be observed too, has taken place wholly in connection with the working years of actual social life. There is no change in the percentage of deaths from this cause in males under 25 years of age. Between 20 and 45 years of age it has risen from 553 to 709, and that almost exclusively in males, for there is almost no increase in the percentage of females dying from heart disease during the twenty-five years of life from 21 to 45. These figures convey their own lesson, and warn us to take a little more care not to kill ourselves for the sake of living.—*British Medical Journal.*

---

THE PHILADELPHIA bogus diploma business has received a well directed blow in the annulling, by the Legislature of Pennsylvania, of the charters of the "Eclectic College of Pennsylvania," and of the "American College,"—better known as the "Philadelphia University of Medicine." Both these institutions had become notorious at home and abroad as dispensers of degrees *in absentia*, and no doubt the illicit and disgraceful traffic in bogus diplomas has been the source of considerable profit. It is creditable to the medical profession in Philadelphia that this action of the State Legislature is largely due to the earnest and continued endeavors of the Faculties of the regular schools to expose the workings of these infamous establishments.—*Boston Med. and Surg. Jour.*

